# Iron Biology in Acute Kidney Injury: Catalytic Iron, Hepcidin–Ferroportin Axis, and NGAL—A Narrative Review

**DOI:** 10.3390/ijms27093802

**Published:** 2026-04-24

**Authors:** Chandrashekar Annamalai, Pragasam Viswanathan

**Affiliations:** Renal Research Lab, Pearl Research Park, School of Biosciences and Technology, VIT, Vellore 632014, Tamil Nadu, India; dr_a_chandrashekar@hotmail.com

**Keywords:** acute kidney injury, catalytic iron, ferroptosis, transferrin, ferritin, hepcidin, ferroportin, NGAL/lipocalin-2

## Abstract

Dysregulated iron handling—including catalytic iron and ferroptosis, hepcidin–ferroportin signaling, ferritin dynamics, and neutrophil gelatinase-associated lipocalin (NGAL)-mediated siderophore transport—has been implicated in the initiation and propagation of acute kidney injury (AKI) across ischemia–reperfusion, sepsis, and nephrotoxic contexts. To provide a SANRA-aligned narrative synthesis of mechanistic and translational evidence on iron biology in AKI, clarifying biomarker readiness and therapeutic prospects while explicitly separating preclinical from human findings. PubMed, Scopus, and Web of Science (1 January 2000 to 30 September 2025), plus appraised grey literature (guidelines/registries) using predefined criteria (authority, update recency, and methodological transparency). Narrative review with comprehensive database searches, single-reviewer screening/extraction (acknowledged as a limitation), and qualitative synthesis. Evidence is organized by pathway (catalytic iron/ferroptosis, transferrin (Tf)/transferrin receptor (/TfR), ferritin/ferritin heavy chain (FtH), hepcidin–ferroportin and NGAL) and translational domain (biomarkers and therapeutics). Statements are tagged as [Preclinical] or [Human]. **[Preclinical]** Robust signals support roles for catalytic iron and ferroptosis, protection by iron chelation, hepcidin modulation, heme oxygenase 1 (HO-1)/FtH induction, and apotransferrin/NGAL-based strategies. **[Human]** Biomarkers such as NGAL show clinical utility for kidney injury detection, whereas catalytic iron assays (labile plasma iron [LPI]/bleomycin-detectable iron [BDI]) remain investigational with limited standardization. Observational links between iron-regulatory pathways and AKI risk exist, but interventional trials are sparse; dose, timing, and safety of iron-targeted strategies in defined AKI settings remain to be established. Iron-handling pathways are compelling targets for AKI prevention/mitigation, yet high-quality human trials are limited. Priorities include standardized catalytic-iron assays, biomarker-guided enrichment, and pragmatic trials of tractable interventions (e.g., peri-contrast or cardiopulmonary bypass settings). Until such evidence accumulates, recommendations beyond standard care should be avoided.

## 1. Introduction

Acute kidney injury (AKI) is common, lethal, and costly worldwide. Iron metabolism and iron-dependent redox biology are increasingly recognized as central to renal pathophysiology, and dysregulated iron handling has been associated with AKI occurrence and worse outcomes [1,2]. In particular, excess catalytic (labile) iron appears mechanistically relevant in settings such as contrast-associated AKI [3].

Iron is indispensable for oxygen transport and cellular energetics—embedded in hemoglobin and myoglobin, in electron-transport chain cytochromes, and as a cofactor for numerous enzymes—thereby participating in many fundamental biological processes [4,5]. The kidneys limit urinary iron losses via tubular reabsorption and express multiple transport and metabolic proteins related to iron, positioning the nephron as an active participant in systemic iron homeostasis [6]. While iron is required for metabolically active renal cells [7], it can also be cytotoxic [8,9]. This duality stems from iron’s redox activity: by mediating electron transfer, iron can catalyze Fenton and Haber–Weiss reactions, generating reactive oxygen species (ROS), including hydroxyl radicals, that overwhelm antioxidant defenses and damage lipids, proteins, and nucleic acids. These processes also promote inflammation and vasoconstriction, contributing to ischemic and toxic injury in the kidney [8].

Concurrently, insights into regulated cell-death programs—especially ferroptosis, an iron-dependent, lipid peroxidation-driven process—have refined our understanding of iron-induced renal cell injury. Emerging work situates ferroptosis alongside oxidative stress, inflammation, and hemodynamic instability as converging mechanisms in AKI pathogenesis [5,10]. Yet important gaps persist; clinical standardization of catalytic iron assays is limited; the readiness level of candidate biomarkers such as neutrophil gelatinase-associated lipocalin (NGAL), labile plasma iron (LPI)/bleomycin-detectable iron (BDI), and hepcidin varies; and interventional human evidence for iron-targeted strategies remains sparse.

**Aim and scope of this review:** We provide a SANRA-aligned narrative synthesis of iron biology in AKI, integrating mechanistic pathways (catalytic iron/ferroptosis; transferrin/TfR; ferritin/FtH; hepcidin–ferroportin; NGAL) with translational implications for biomarkers and therapies. To maintain interpretability and clinical relevance, we explicitly tag evidence as preclinical (in vitro/animal) or human and avoid efficacy inferences where clinical data are lacking.

## 2. Methods

### 2.1. Review Design and Objectives

This work is a narrative review conducted in line with SANRA items (aims justified; literature search described; appropriate referencing; scientific reasoning; added value). Objectives were to (a) summarize the pathophysiology of iron-related injury in AKI (catalytic/labile iron, ferroptosis, transferrin/transferrin receptor (TfR), ferritin/ferritin heavy chain (FtH), hepcidin–ferroportin and NGAL) and (b) synthesize recent advances in prevention and treatment of renal ferrotoxicity.

### 2.2. Eligibility Criteria

- **Population/Models:** Human adults >18 years experiencing AKI of any etiology; relevant preclinical models (non-human primates, mice, and in vitro systems).
- **Exclusions:** Pediatric studies (≤18 years), chronic kidney disease and kidney-transplant studies unless directly informing AKI mechanisms/translation, and articles clearly outside the conceptual scope.
- **Language:** English. (Acknowledged as a limitation.)

### 2.3. Information Sources

Potentially relevant documents, including peer-reviewed journal articles, systematic reviews, meta-analyses, e-books, theses, dissertations, letters, guidelines, websites, blogs, and conference materials, written in English, were identified by searching the following bibliographic databases from January 2000 to September 2025: MEDLINE (PubMed), Scopus, and Web of Science. Searches were designed to capture both human and preclinical evidence (including non-human primates, murine models, and in vitro studies). In PubMed, population filters were applied at the database search stage (humans > 18 years OR non-human primates OR mice OR in vitro) to retrieve both adult human and preclinical records; pediatric human studies were excluded at search and confirmed during screening (title/abstract/full text). Language was restricted to English. Equivalent database-level age filtering was not applied in Scopus and Web of Science due to platform constraints; pediatric human records from these databases were excluded during screening (title/abstract/full-text). Supplementary approaches included checking reference lists of included or relevant sources and targeted searches of trial registries or regulatory websites. Any missing or unpublished information was sought by contacting authors or sponsors when necessary. Search strategies were drafted and refined through team discussion. Duplicates were removed after exporting the final search results for de-duplication using EndNote.

### 2.4. Timeframe

Searches were run on **11 October 2025** and covered the period **1 January 2000 to 30 September 2025.**

### 2.5. Search Strategy

Strategies combined controlled vocabulary (where available) and free-text terms for **iron biology** (catalytic/labile iron, ferroptosis, transferrin, ferritin, hepcidin, ferroportin, NGAL/LCN2) and **acute kidney injury**. A representative Boolean framework (PubMed-style) is shown below (as well as in Supplementary File S1), mirroring the conceptual chart used during screening.


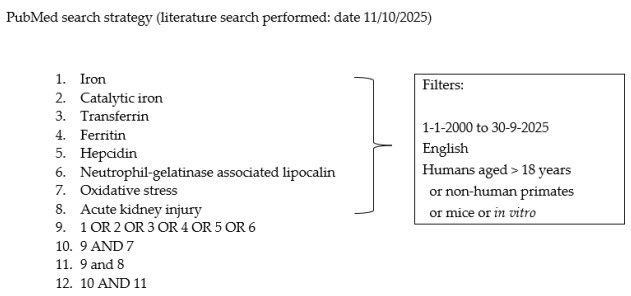


Database-specific expansions (MeSH/Keywords) were applied as appropriate; complete strings for each database are provided in the Supplementary File S2 to ensure reproducibility.

A brief search-yield summary is provided below:

PubMed: **427**; Scopus: **2601**; Web of Science: **1093**; other sources: **28**; total retrieved: **4149**; duplicates removed: **1469**; records after deduplication: **2670**. Of 2670 unique records after deduplication, 2133 were excluded as off-scope at title/abstract screening; 357 pediatric human records (primarily retrieved via Scopus/Web of Science searches that did not permit an age filter) were excluded at screening because this review focused on adult AKI, and pediatric AKI differs in etiologies/exposures and biomarker behavior/thresholds; 18 records with missing/invalid bibliographic metadata (e.g., publication year) were removed because reliable screening was not possible; and 3 full texts could not be retrieved (inaccessible/behind paywall). Thus, 159 studies were retained and contributed to the narrative synthesis (2670 − (2133 + 357 + 18 + 3) = 159).

### 2.6. Selection of Sources of Evidence

A single reviewer conducted an unblinded eligibility assessment using a standardized guide discussed with a supervisor prior to screening. Titles/abstracts/full texts were reviewed against the criteria above. This single-reviewer process is acknowledged as a limitation.

### 2.7. Data Charting/Extraction

A structured electronic form (adapted from Cochrane data-collection guidance) [11] captured study design; species/model (for preclinical) or human cohort characteristics; AKI context (e.g., contrast, cardiopulmonary bypass (CPB), ischemia-reperfusion injury (IRI), sepsis, nephrotoxins); exposure/intervention; biomarkers/assays (e.g., NGAL, LPI/BDI, hepcidin); outcomes; effect direction; and evidence tier (preclinical vs. human). Disagreements/uncertainties were resolved through discussion with the supervisor.

### 2.8. Synthesis of Data

Studies were grouped by pathway (catalytic iron/ferroptosis; transferrin; ferritin/HO-1; hepcidin–ferroportin; NGAL) and translational domain (biomarkers, therapeutics). When a systematic review was identified, we recorded its included study count relevant to our criteria and noted any additional studies our search retrieved. Findings are presented narratively with explicit evidence-tier tagging; no meta-analysis or formal risk-of-bias tool was applied (narrative design), though methodological strengths/weaknesses are summarized qualitatively in the ensuing discussion.

## 3. Pathophysiology of Iron-Induced AKI

Changes in iron processing and iron-induced cytotoxicity have been investigated as both causes and consequences of renal injury over several decades [12]. We summarize key mechanisms and evidence below, explicitly tagging **[Preclinical]** (in vitro/animal) and **[Human]** data.

### 3.1. Iron-Induced Oxidative Stress and Endoplasmic Reticulum Stress

**Figure 1:**
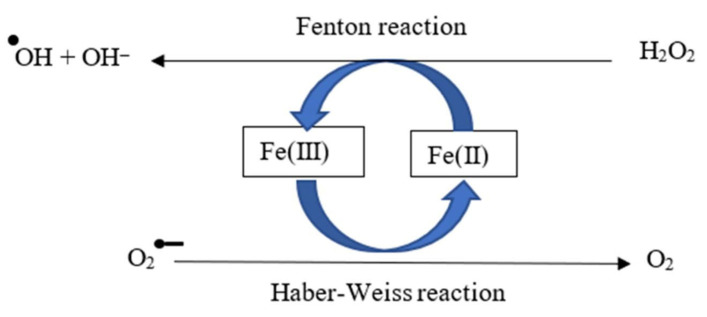
Reactive oxygen species production by catalytic iron through reversible cycling between ferric and ferrous oxidation states. ·OH·, Hydroxyl radical; OH^−^, Hydroxy group; H_2_O_2_, Hydrogen peroxide; O_2_^−^, Superoxide anion.

Reactive oxygen species (ROS)—free radicals such as superoxide (O_2_^−^) and hydroxyl (·OH) and non-radicals such as hydrogen peroxide (H_2_O_2_) and peroxynitrite (ONOO^−^) [13]—oxidize proteins and lipids, damage DNA [14], depolarize mitochondria, and trigger apoptosis [15], thereby mediating cytotoxicity, inflammation, and vasoconstriction [8]. Ferrous iron catalyzes Fenton chemistry [16] and, together with superoxide/H_2_O_2_ participates in the Haber–Weiss sequence to generate ·OH [17], promoting lipid peroxidation and cell injury (Figure 1).

**[Preclinical]** ROS-iron interactions are well demonstrated in animal models [18,19]. Paller (1988) showed lipid peroxidation driven by free and heme iron in rat AKI from glycerol, hemoglobin, and ischemia [20]. Iron-dependent mechanisms have been implicated in renal ischemia–reperfusion injury (IRI) [21], and hydroxyl radical involvement was supported in glycerol-induced AKI models [22]. Furthermore, endoplasmic reticulum (ER) stress commonly accompanies renal injury and can be directly induced in tubular epithelial cells by exposure to iron or heme [23,24].

### 3.2. Iron-Induced Kidney Injury

Systemic iron overload, enhanced tubular iron delivery, or disturbed intracellular localization can raise transferrin-bound and labile/catalytic iron pools. **[Preclinical]** Direct tubular cytotoxicity from iron has been shown in vitro [25,26] and in vivo [27,28], and excess renal/urinary iron has been observed in **[Human]** patients [29,30] and **[Preclinical]** animal models [31,32] of AKI. Dietary iron restriction, chelators, or antioxidant strategies mitigate the intensity of renal injury [33,34,35].

Inflammation/oxidative stress upregulates iron-regulatory proteins—DMT1, Zip8/Zip14, lactoferrin, hepcidin, and NGAL—enhancing parenchymal iron uptake [36,37,38,39]. While sequestration may be adaptive, severe inflammation/oxidative stress can drive pathologic accumulation and ferrotoxicity. Renal cells are sensitive to ferroptosis, an iron-dependent, lipid-peroxidation–driven cell death involving damage-associated molecular patterns (DAMPs) and reactive organelles such as lysosomes, peroxisomes, and mitochondria. This process is followed by an immunological response activating other cell death pathways, including necroptosis, which is induced by inflammation alone, and pyroptosis, induced by both inflammation and oxidative stress [40,41,42,43,44].

**Figure 2:**
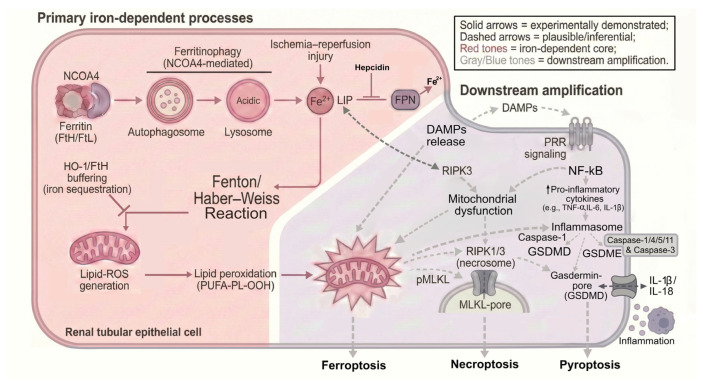
Integration of primary iron-dependent processes (left, red tones) and downstream inflammatory amplification (right, gray/blue tones) in a renal tubular epithelial cell. The schematic illustrates mitochondrial dysfunction and iron-dependent ROS as a common denominator for crosstalk between regulated cell death pathways. **Primary Phase (Core Injury):** NCOA4-mediated ferritinophagy delivers ferritin (FtH/FtL) to lysosomes, releasing Fe^2+^ and expanding the labile iron pool (LIP). This excess Fe^2+^ fuels Fenton/Haber–Weiss chemistry and lipid-ROS generation, driving phospholipid peroxidation (PUFA-PL-OOH) and ferroptosis. This core injury is modulated by iron-handling pathways: hepcidin increases toxicity by inhibiting ferroportin (FPN)-mediated export, whereas sequestration by FtH and HO-1 removes catalytic iron and inhibits Fenton chemistry. **Downstream Phase (Amplification and Cell Death):** Intracellular iron overload and the resulting oxidative environment facilitate the activation of the necrosome (RIPK1/RIPK3 complex), executing necroptosis via pMLKL pore formation. Injury is further amplified by the release of damage-associated molecular patterns (DAMPs), which engage pattern-recognition receptor (PRR) signaling to activate NF-κB. This signaling induces proinflammatory cytokines (TNF-α, IL-6, and IL-1β) and activates the inflammasome/caspase-1 axis, culminating in pyroptosis via GSDMD/GSDME gasdermin pores (where GSDME cleavage involves caspase-3). Legend: Solid arrows = experimentally demonstrated links; dashed arrows = plausible/inferential connections; T-bar = inhibition line. Abbreviations: DAMP, damage-associated molecular pattern; FPN, ferroportin; FtH/FtL, ferritin heavy/light chain; GSDMD/E, gasdermin D/E; HO-1, heme oxygenase-1; IL, interleukin; LIP, labile iron pool; NCOA4, nuclear receptor coactivator-4; NF-κB, nuclear factor kappa-light-chain-enhancer of activated B cells; pMLKL, phosphorylated mixed-lineage kinase domain-like; PRR, pattern-recognition receptor; RIPK, receptor-interacting protein kinase; TNF-α, tumor necrosis factor-alpha.

Figure 2 illustrates necroptosis proceeding via its canonical pathway, where signaling involving Receptor-Interacting Protein Kinase 3 (RIPK3) leads to the subsequent phosphorylation of Mixed Lineage Kinase Domain-Like pseudokinase (pMLKL) [45]. Phosphorylated MLKL then oligomerizes and executes membrane disruption through the formation of MLKL-pores at the plasma membrane [46].

Furthermore, the figure indicates inferential crosstalk between the ferroptotic and necroptotic pathways [47]. Ferroptosis-induced mitochondrial dysfunction is suggested to communicate with downstream amplification pathways, including necroptosis [48]. Additionally, upstream signaling components like RIPK3 may potentially link to the primary iron-dependent processes by influencing the Labile Iron Pool (LIP) [47,49].

### 3.3. Role of Catalytic Iron

Catalytic (labile) iron is a transitional, largely ferrous iron (Fe^2+^) pool not bound to transferrin and loosely complexed to albumin and low-molecular-weight ligands [50]. This redox-active pool can drive oxidative injury; the underlying Fenton/Haber–Weiss chemistry is described in Section 2.1 [8,15]. Catalytic iron may also promote **prothrombotic** states resistant to fibrinolysis [51,52].

#### 3.3.1. Sources of Catalytic Iron

Under physiological conditions, transferrin and ferritin buffer circulating iron, limiting detectable catalytic iron. Metabolic acidosis, hemolysis/hemorrhage, or release from intracellular stores during tissue injury can overwhelm buffering and elevate circulating labile iron [15,53]. **[Preclinical]** Excess renal iron during acute injury has been traced to degraded erythrocytes (hemolysis) [18] or rhabdomyolysis [53]. Stored packed red blood cells (pRBCs) develop storage lesions with pro-inflammatory mediators [54] and hemolysis [55], and **[Human]** studies note markedly elevated labile iron in pRBC aliquots with longer storage [56], alongside observational links between older pRBCs and AKI after cardiac surgery [57]. Additional sources include ferritin-mediated iron release [58,59], mitochondrial iron [60,61], and free heme [62]. **[Preclinical]** Gentamicin-induced AKI implicated mitochondrial iron; catalase conferred renoprotection even when other scavengers/chelators were used [60]. Cisplatin reduces cytochrome P450 with a concomitant rise in bleomycin-detectable iron; P450 inhibition through piperonyl butoxide mitigates injury [63].

#### 3.3.2. Determination of Catalytic Iron

Catalytic (labile) iron is commonly assessed using the bleomycin-detectable iron (BDI) assay and the labile plasma iron (LPI) assay. BDI quantifies DNA damage generated by bleomycin in the presence of catalytic iron and a reducing agent, whereas LPI quantifies oxidation of dihydrorhodamine to a fluorescent product and uses a chelator-inhibitable signal to indicate chelatable iron [8,59,60,64,65].For clinical translation, standardized cut-offs are not established, and inter-assay comparability remains limited, constraining risk stratification and cross-study pooling [66,67,68,69]. Pre-analytical variables (e.g., anticoagulant choice), handling and storage, and timing relative to injury onset materially affect BDI/LPI results, limiting cross-study comparability and precluding routine clinical deployment at present.

#### 3.3.3. Catalytic Iron—In-Vitro Studies

**[Preclinical]** Myoglobin loading increases catalytic iron and predisposes proximal tubular segments isolated from male Sprague Dawley rats to H_2_O_2_-mediated injury [70], although myoglobin can paradoxically protect against Fe-mediated damage absent iron release from the porphyrin ring [62]. In glycerol AKI, intrarenal myoglobin reduced cortical malondialdehyde levels unexpectedly [71], underscoring context dependence. Fe^2+^ and Fe^3+^ salts show concentration-dependent cytotoxicity in Vero cells, with Fe^2+^ more toxic (greater proliferation inhibition and a lower EC50, the half maximal effective concentration using the dose-response curve) [72]. A similar higher in vivo toxicity of Fe^2+^ has been reported [73].

#### 3.3.4. Catalytic Iron—Animal Studies

**[Preclinical]** Across models, elevated labile iron is observed, and chelation mitigates structural and functional injury:

- **Myohemoglobinuric kidney injury:** Glycerol rat models show increased catalytic iron and reduced cytochrome P450; P450 inhibitors (cimetidine, piperonyl butoxide) lower iron [53]. Hydroxyl radical scavengers such as dimethylthiourea (DMTU) and deferoxamine are protective [22], consistent with earlier work [20,74].
- **Cisplatin nephrotoxicity:** BDI increases after cisplatin; deferoxamine improves BUN/creatinine and histology [19]. P450 inhibition attenuates catalytic iron rise and injury [63].
- **Aminoglycoside nephrotoxicity:** Gentamicin plus DMTU (·OH scavenger) or deferoxamine lowers BUN and malondialdehyde and improves histology [75]; gentamicin mobilizes mitochondrial iron [60].
- **Contrast-induced nephropathy:** Deferoxamine pretreatment reduces CI-AKI in rabbits [76]; oxidative injury likely mediates toxicity [77].
- **Ischemia–reperfusion injury:** Reperfusion (not ischemia) is associated with increased renal catalytic iron in rats [18], with corroborating rabbit data [78]. Chelators (deferoxamine and apotransferrin) and NGAL infusion mitigate injury [34,35,79,80]; iron-saturated transferrin does not [35]. Benefits are attributed to binding/eliminating catalytic iron and, for NGAL, potential HO-1 upregulation.

#### 3.3.5. Catalytic Iron—Human Studies

**[Human]** Direct renal tissue sampling is rare; most studies rely on serum/urine labile iron [81].

- **Systemic iron overload:** Kidney involvement in hemochromatosis is recognized [82,83]. Tubular dysfunction occurs with iron-rich diets (mice) [84] and transfusion-related iron loading (patients) [85,86]. Chronic hemolysis/inflammation may stimulate hepatic and renal hepcidin [38], promoting iron accumulation and injury [87].
- **Glomerulopathy/hematuria:** Renal iron deposition is associated with proteinuria [88] and hematuria [29], with tubular injury [29] and urinary iron loss reported in animals and patients [32,89,90,91]. Iron restriction/chelation reduced proteinuria [91,92] and hypertension [33,93] and improved renal function in experimental/clinical contexts [94]. Sources implicated include catalytic iron from injured glomerular cells [91], transferrin-bound iron [25], and free hemoglobin [26,29].
- **Heme-related AKI:** Hemolysis and rhabdomyolysis overwhelm haptoglobin/hemopexin, increasing filtered heme [31,95]. **[Human/Preclinical]** High urinary iron accompanies hemolytic diseases [30] and heme-mediated injury [71], with tissue injury despite upregulated HO-1, ferritin, ferroportin, haptoglobin, hemopexin, and CD163 [31,96,97]. Histology may show hemosiderin, heme casts, tubular necrosis, cortical atrophy, and interstitial fibrosis [29,31]. Heme-protein associations with AKI are well described [98].
- **Cardiopulmonary bypass (CPB)–associated AKI:** Hemolysis from non-physiologic surfaces/shear and muscle injury (myoglobin) can elevate free hemoglobin and labile iron; transfusions and IRI contribute [55,99,100]. Elevated free hemoglobin correlates with labile iron, with higher catalytic iron linked to AKI, mortality, and myocardial injury after age- and pre-operative estimated glomerular filtration rate (eGFR) adjustment [56]. Urinary catalytic iron correlates with NGAL early after CPB [99].
- **Ischemia–reperfusion injury:** Mechanistically, ischemia lowers pH or reduces Fe^3+^ to Fe^2+^, releasing cytosolic protein-bound iron; reperfusion augments delivery of circulating and liberated iron, driving injury [34,79,80].
- **Contrast-induced AKI:** In ACS cohorts, elevated catalytic iron associates with mortality (e.g., OPUS-TIMI 16) [101] and rises after contrast in those developing CIN, with higher baseline catalytic iron predicting adverse outcomes [3].
- **Critical illness–associated AKI:** Elevated plasma catalytic iron in intensive care unit (ICU) patients is associated with incident AKI, dialysis, and mortality after adjustment for age, eGFR, and transfusion [102].

In a nutshell, higher catalytic iron was associated with mortality, and where reported, this persisted after multivariable adjustment. Nevertheless, residual confounding, reverse causality, and co-factors (e.g., illness severity, transfusions, inflammation, and baseline kidney function) cannot be excluded. For an at-a-glance summary of contexts, assays/biomarkers, and direction of findings across preclinical and human studies, see Table S1 (Supplementary Materials).

**Translational summary:** Evidence across settings links catalytic iron to AKI risk/severity. While preclinical data suggest potential for chelation, apo-transferrin, and NGAL-based strategies, confirmatory human trials are limited. Because LPI/BDI assays lack standardized cutoffs and cross-platform comparability, assay harmonization is a prerequisite for clinical deployment [66,67]. For future studies, we outline trial-ready contexts (CPB/contrast and cisplatin) and biomarker-guided enrichment (NGAL for injury detection and LPI/BDI for iron burden) with standardized endpoints (Kidney Disease: Improving Global Outcomes [KDIGO] AKI within 48–72 h and MAKE30—composite of death, new renal replacement therapy (RRT), or persistent kidney dysfunction at 30 days) [68,69,103,104].

**Figure 3:**
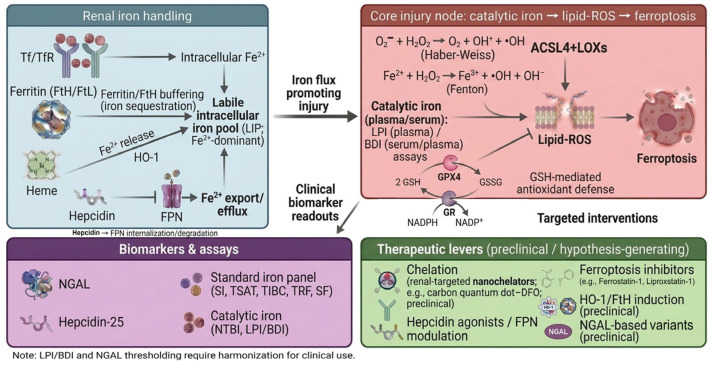
Overview map of iron biology → biomarkers/therapeutics. Renal iron handling (left) shapes the labile intracellular iron pool (LIP; Fe^2+^-dominant) through transferrin/transferrin receptor (Tf/TfR)–mediated uptake, ferritin/ferritin heavy chain (FtH) buffering (iron sequestration), heme oxygenase-1 (HO-1)–linked Fe^2+^ release, and ferroportin (FPN)–mediated Fe^2+^ export/efflux (hepcidin promotes FPN internalization/degradation). Increased iron flux converges on a core injury node (center) in which circulating catalytic iron (plasma/serum; labile plasma iron [LPI] and bleomycin-detectable iron [BDI] assays) drives Fenton/Haber–Weiss chemistry, lipid reactive oxygen species (lipid-ROS) generation (acyl-CoA synthetase long-chain family member 4; ACSL4 + lipoxygenases; LOXs), and ferroptosis, counterbalanced by glutathione peroxidase 4 (GPX4) antioxidant defense. Specifically, GPX4 utilizes reduced glutathione (GSH) to detoxify lipid hydroperoxides, a process that oxidizes GSH to glutathione disulfide (GSSG). To sustain this antioxidant defense, glutathione reductase (GR) regenerates GSH from GSSG using NADPH as an electron donor. Clinical biomarker and assay readouts (bottom-left) include neutrophil gelatinase–associated lipocalin (NGAL), hepcidin-25, standard iron panel indices (serum iron [SI], transferrin saturation [TSAT], total iron-binding capacity [TIBC], transferrin [TRF], serum ferritin [SF]), and catalytic iron measures (non-transferrin-bound iron [NTBI], LPI/BDI). Therapeutic levers (bottom-right; preclinical/hypothesis-generating) include chelation (including renal-targeted nanochelators; e.g., carbon quantum dot–deferoxamine [DFO]), apotransferrin (apo-Tf), hepcidin agonists/FPN modulation, ferroptosis inhibitors (e.g., liproxstatin-1), HO-1/FtH induction, and NGAL-based variants. Note: LPI/BDI and NGAL thresholds require assay harmonization for clinical use. And the protein representations (e.g., Tf, ferritin, NGAL) are schematic symbols intended to illustrate functional nodes and do not depict high-resolution structural models or atomic conformations.

Taken together, the evidence above positions catalytic (labile) iron as a mechanistic hub linking upstream iron-handling pathways to oxidative injury, lipid peroxidation, and ferroptosis, while also highlighting where measurements and interventions can be grounded. To help readers transition from pathway biology to measurable biomarkers/assays and hypothesis-generating therapeutic levers discussed in the following sections, Figure 3 provides an overview map of renal iron handling → catalytic iron/ferroptosis node → clinical biomarker/assay readouts and candidate intervention points.

### 3.4. Role of Transferrin (Tf) in AKI

Serum transferrin (Tf) is the principal physiological iron chelator in plasma. Each monomeric glycoprotein (~76–81 kDa) has two Fe^3+^-binding sites (N- and C-lobes) [105], binding iron avidly and reversibly to maintain Fe^3+^ solubility, regulate cellular delivery, and—critically—lower the redox potential of iron, thereby limiting oxygen-radical formation. By restricting iron availability, Tf also contributes to nutritional immunity against pathogens [106] and participates in hepcidin regulation [107].

Transferrin saturation (TSAT) reflects iron-loading status; ~30% saturation is typical physiologically. When iron overload drives TSAT > 60%, non-transferrin-bound iron (NTBI) rises, markedly increasing redox-active iron and tissue accumulation/injury [108].

Cellular uptake occurs via transferrin receptor 1 (TfR1), widely expressed, wherein holo-Tf/TfR1 complexes undergo endocytosis; Tf’s plasma half-life is ~8 days, and cellular distribution via Tf/TfR1 occurs over minutes [109]. Transferrin receptor 2 (TfR2), predominant in hepatocytes and with ~25-fold lower affinity for holo-Tf than TfR1, is implicated primarily in hepcidin regulation [110]. In the kidney, transferrin-bound iron is filtered and reabsorbed by megalin–cubilin on the apical membrane of proximal tubular cells, facilitating cellular iron handling [82].

**Translational context (mechanistic plausibility vs. gaps):**

- **[Preclinical]** Apo-transferrin (iron-free Tf) limits delivery of redox-active iron and shows a signal of renoprotection in ischemia–reperfusion–like models, consistent with reduced catalytic-iron burden and improved structural/functional readouts (See Section 3.3.4 and Section 3.3.5).
- **[Human]** Direct interventional evidence that modulating Tf or administering apo-Tf improves clinical AKI outcomes is limited; feasibility, timing, and dosing remain open questions and should be addressed in targeted trials. However, observational cardiac surgery datasets suggest context-dependent, non-linear associations between iron transport indices and cardiac surgery–associated AKI: in a two-dataset retrospective analysis (Fuwai Hospital, *n* = 744; Medical Information Mart for Intensive Care IV [MIMIC-IV], *n* = 744), restricted cubic spline analyses showed higher AKI risk with transferrin (Tf) > 2.43 g/L (as reported), TSAT < 31%, and TIBC > 54 μmol/L (J-shape) in Fuwai, while in MIMIC-IV serum ferritin (SF) showed a J-shape (higher risk beyond 143 μmol/L, as reported) and serum iron (SI) a reverse U-shape—consistent with non-linear risk across iron deficiency and surplus states, potentially modified by peri-operative hemolysis/oxidative stress [103].

**Assay/biomarker note:** While Tf and TSAT are routine, combining them with LPI/BDI to quantify catalytic iron burden is limited by the lack of standardized cut-offs and cross-platform assay comparability [66,67,68,69].

### 3.5. Role of Ferritin in AKI

Ferritin is the principal intracellular iron-storage protein, composed of heavy (FtH) and light (FtL) chains. FtH has ferroxidase activity that converts Fe^2+^ → Fe^3+^ for safe mineralization within the ferritin core; a single ferritin shell can sequester up to ~4500 Fe atoms, conferring potent endogenous iron-chelating capacity [111]. FtL lacks ferroxidase activity but facilitates nucleation/mineralization of the iron core [112].

Both subunits are expressed in the kidney [82,113], acting cooperatively to buffer labile iron according to metabolic needs. **[Preclinical]** Oxidant stress can enhance iron release from storage proteins while simultaneously inducing ferritin up-regulation—an adaptive response that sequesters free iron and limits ferrotoxicity [114]. In proximal tubular FtH knockout mice, kidney injury triggers pronounced pro-inflammatory macrophage infiltration, cytokine production, fibrosis, and tubular–macrophage crosstalk [115]; these mice also exhibit increased tubular apoptosis and mortality after rhabdomyolysis and cisplatin exposure, underscoring FtH’s cytoprotective role [113].

**[Human]** Perioperative data are mixed: a small cardiac-surgery pilot linked lower pre-operative ferritin with higher AKI risk [116], but a larger extension did not confirm the association [117]; although residual confounding and reverse causality cannot be excluded despite adjustment.

**Double-edged biology:** Although ferritin is a storage protein, it can release iron under inflammatory/oxidative conditions (e.g., superoxide-driven release) [58], potentially fueling labile-iron pools; thus, ferritin may function as either a sink or a source of catalytic iron depending on redox context [56].

**Translational context (mechanistic plausibility vs. gaps):** Strategies that induce FtH/HO-1 or augment ferritin buffering are biologically plausible for limiting ferrotoxicity; however, human interventional evidence remains limited, and efficacy should not be inferred beyond preclinical/observational signals. Consistent with this, recent preclinical work in a murine renal ischemia–reperfusion injury model reported that silibinin improved kidney function and histology, lowering blood urea nitrogen (BUN), serum creatinine, tubular injury scores, and neutrophil gelatinase–associated lipocalin (NGAL)/kidney injury molecule-1 (KIM-1); post-injury dosing retained benefit, though attenuated. Mechanistically, silibinin inhibited ferroptosis in vivo and in renal tubular cells (human HK-2, rat NRK-52E, and primary mouse proximal tubular epithelial cells), reducing lipid peroxidation (malondialdehyde, MDA); restoring glutathione (GSH) and superoxide dismutase (SOD) limiting lipid reactive oxygen species; and decreasing ferrous iron (Fe^2+^) and Prussian-blue–detectable iron. Proteomic/biophysical assays (molecular docking, surface plasmon resonance imaging, cellular thermal shift, and drug affinity responsive target stability) supported direct binding to ferritin heavy chain (FTH1) and disruption of the NCOA4–FTH1 interaction, suppressing ferritinophagy; FTH1 knockdown attenuated protection, indicating FTH1 dependence. Collectively, these data support FtH-centered strategies to buffer labile iron and blunt ferroptosis in AKI, while remaining preclinical and hypothesis-generating for human translation [118].

### 3.6. Role of Hepcidin in AKI

Hepcidin-25 (LEAP-1), encoded by HAMP, is a cysteine-rich antimicrobial peptide produced mainly by the liver as an 84-amino acid prepropeptide and processed to the active 25-amino acid isoform with a specific N-terminus [119]. Of the circulating isoforms (hepcidin-20/-22/-25), hepcidin-25 is the functional ligand for ferroportin, the sole known cellular iron exporter. Hepcidin circulates largely bound to α2-macroglobulin, is freely filtered at the glomerulus, and is ~97% reabsorbed in proximal tubules via megalin-mediated endocytosis. Renal epithelial expression of hepcidin mRNA in cortex and medulla suggests local renal synthesis [120].

**Mechanism and regulation:** Hepcidin negatively regulates intestinal iron absorption and iron egress from enterocytes, hepatocytes, and macrophages by binding ferroportin, triggering its internalization and ubiquitin-mediated degradation [121]. Hepcidin is upregulated by iron loading and inflammation and downregulated by hypoxia and anemia/erythropoietic drive [122]. In the kidney, hepcidin can confer renoprotection by sequestering intracellular iron (limiting oxidative injury) [123] and by inducing H-ferritin [124].

**Evidence base:**

- **[Preclinical]** Hepcidin expression increases with systemic iron overload (liver upregulation) [125], and exogenous hepcidin or mimetics reduce the severity of ischemia–reperfusion and hemoglobin-mediated renal injury in rodent models [124,126].
- **[Human]** Observational data suggest higher perioperative urinary hepcidin associates with lower AKI risk after cardiac surgery [123,127], and increased hepcidin correlates with better survival in severe illness requiring dialysis [128]. However, residual confounding and reverse causality remain possible despite adjustment.

**Assays and translational considerations:** Hepcidin can be measured by mass spectrometry–based methods or immunoassays; inter-assay variability and isoform specificity limit cross-study comparability. Hepcidin agonists/mimetics (e.g., minihepcidins, rusfertide) are in clinical evaluation for other iron-disorder indications, but their use in AKI remains hypothesis-generating and requires targeted trials [129,130,131].

**Clinical balance:** Excessive hepcidin activity can promote functional iron deficiency and anemia; any interventional strategy must balance potential renoprotection against systemic effects on erythropoiesis and infection risk. Trial designs should prioritize biomarker-guided enrichment (e.g., high free hemoglobin (Hb)/labile iron states such as CPB and rhabdomyolysis) and incorporate standardized hepcidin and catalytic iron measurements.

### 3.7. Role of Ferroportin (FPN1/SLC40A1) in AKI

Ferroportin (FPN1; gene: SLC40A1) is a multi-pass membrane iron exporter with 12 transmembrane (TM) helices and a prominent cytosolic loop between TM6 and TM7; both N- and C-termini are cytosolic. Hepcidin-25, its sole known ligand, binds FPN and interacts critically with Cys326, promoting conformational change, internalization, and lysosomal degradation [132].

**Localization and regulation:** FPN1 is ubiquitous. In the kidney, it is detected predominantly on basolateral membranes of proximal tubular epithelial cells (facilitating iron efflux to the interstitium/circulation), with reports of apical/brush-border presence and cytoplasmic pools under certain conditions. Expression is dynamically regulated at transcriptional, translational, and post-translational levels and, together with hepcidin, forms the core of systemic iron homeostasis by modulating duodenal absorption, macrophage iron efflux, and hepatic storage [133]. In iron-overload states, apical up-regulation of FPN1 on proximal tubules has been observed, consistent with attempts to eliminate excess iron in urine [134].

**Implications for AKI:**

- **[Preclinical]** Modulation of the hepcidin–ferroportin axis alters susceptibility to ischemia–reperfusion and hemoglobin-mediated injury in rodents (see Section 2.6), supporting a role for controlled iron sequestration versus efflux in limiting catalytic iron–driven damage.
- **[Human]** Direct evidence linking renal FPN1 expression patterns to AKI outcomes is limited; small studies and indirect markers (hepcidin, TSAT, LPI/BDI) suggest that dysregulated export may contribute to labile iron accumulation.

**Translational consideration:** Therapeutic strategies targeting this axis include hepcidin agonists/mimetics [129,130] and potentially ferroportin inhibitors/antagonists; however, risks include hypoferremia and anemia. Any interventional approach in AKI should be biomarker-guided and context-specific (e.g., hemolysis-dominant states) [135].

### 3.8. Role of NGAL (LCN2) in AKI

Neutrophil gelatinase-associated lipocalin (NGAL; LCN2) is a ~25-kDa lipocalin that binds bacterial and fungal siderophores, sequestering labile iron and exerting antimicrobial effects [136]. In the kidney, NGAL is produced by proximal and distal tubular cells (as well as neutrophils/macrophages) and appears in plasma and urine. NGAL can bind urinary catalytic iron, while unbound labile iron may be reabsorbed by nephron segments (e.g., thick ascending limb, cortical collecting duct) [137].

**Diagnostic/Prognostic biomarker role: Urinary/plasma** NGAL is recognized as an early marker of kidney injury with broad validation across ischemic, septic, and nephrotoxic AKI contexts [138]. Microarray and transcript studies show early NGAL up-regulation after ischemia [139].

**Intrarenal biological effector role: [Preclinical]** Raising circulating NGAL before injury mitigates functional and structural kidney damage [139,140], and NGAL knockout mice exhibit worse injury (e.g., glomerular damage/proteinuria), consistent with anti-inflammatory effects [141]. NGAL’s protective actions may be partly HO-1 dependent [142]. In this context, it is important to distinguish mechanism from clinical interpretation: while NGAL upregulation is biologically protective, in human studies, elevated urinary NGAL primarily reflects the magnitude of tubular injury rather than a successful mitigation of damage. **Clinical complexity and conflicting signals:** Despite its protective potential, NGAL has been associated with worsening progressive CKD in some settings [143], possibly via pro-inflammatory signaling and iron mobilization [136]. After cardiopulmonary bypass (CPB), higher urinary NGAL correlates with greater AKI risk [144], which may reflect injury burden rather than causality; importantly, urinary NGAL does not necessarily mirror local tissue NGAL levels, and CPB’s inflammatory milieu could override NGAL’s cytoprotective effects [6].

**Assay harmonization and thresholds:** NGAL is measured across multiple platforms and specimen types (plasma vs. urine), but standardized clinical cut-offs and cross-platform harmonization are not yet established, limiting universal implementation [145,146]. Recent synthesis work reinforces these constraints: across intensive care unit, perioperative, and nephrotoxin-associated settings, urine and plasma NGAL remain among the earliest-rising AKI biomarkers, but reported cut-offs vary widely by assay platform (e.g., Architect, Triage, enzyme-linked immunosorbent assay) and by matrix (urine vs. plasma), limiting generalizability and supporting the need for context-specific thresholds when combining NGAL with creatinine in risk models [146]. In addition, a systematic review in intensive care unit adults (2015–2025) reported good early diagnostic performance (notably higher sensitivity within 0–12 h vs later windows) but substantial heterogeneity by cut-off, timing, and platform; the authors recommend standardized sampling windows, platform-specific calibration, and reporting of prespecified cut-offs to improve clinical translation [147].

**Experimental therapeutics (Engineered NGAL variants; preclinical only):** Engineered NGAL variants have been described in preclinical contexts for siderophore/iron modulation [148]; **no human** interventional data are available to date.

## 4. Prevention and Treatment of Iron-Induced AKI

Advances in renal iron-handling biology under physiological and pathological states have spurred investigation into preventive and therapeutic concepts to curb ferrotoxicity [6,12]. Below we summarize key approaches with evidence-tier tagging, noting that currently these strategies are biologically plausible but largely hypothesis-generating rather than clinically established.

### 4.1. Iron Chelators

**[Preclinical]** Classical chelators—Ethylenediaminetetraacetic acid (EDTA), deferoxamine (DFO), and deferasirox (DFX)—have been observed to attenuate AKI severity across models by binding circulating (and, to a lesser extent, intracellular) iron and promoting elimination, with reported improvements in biochemical injury and histology (glycerol, cisplatin, and IRI) [20,22,79]. **[Human]** Deferiprone (DFP), another iron chelator, has also been noted to have potential utility in patients with diabetic and non-diabetic glomerular diseases [94]. **[Innovation]** Kidney-targeted nanoplatforms (e.g., carbon quantum-dot drug conjugates carrying DFO) exhibit dual activity—iron scavenging plus ROS reduction—with favorable renal biodistribution in chemotherapy-associated AKI models; however, clinical applicability remains speculative [149].

### 4.2. Antioxidants

**[Preclinical]** Free-radical scavengers (e.g., dimethyl thiourea, sodium benzoate), superoxide dismutase, and allopurinol (xanthine oxidase inhibitor) appear to attenuate nephrotoxicity in animal models [19,22]. Emerging lines extend beyond classical scavengers: small-molecule lipid-peroxyl radical traps, repurposed agents that blunt lipid peroxidation and iron accumulation (e.g., entacapone), and nanoplatforms with renal retention that couple **reactive oxygen species (ROS)** quenching with iron handling (e.g., mackinawite nanoenzymes; Fe–curcumin coordination polymer nanodots) suggest protective potential in AKI models by reducing lipid peroxidation and oxidative injury [104,150,151].

**[Human]** To date, antioxidant strategies have not demonstrated consistent clinical benefit. Contrary to expectations, robust clinical benefit has not been demonstrated, with heterogeneous dosing, timing, and targets likely contributing to negative or inconclusive results [152].

**Translational notes:** Given redox pleiotropy and potential off-target effects, antioxidant strategies are currently best conceptualized as adjuncts within biomarker-guided trials (e.g., paired with catalytic-iron burden measures and injury markers) rather than stand-alone therapies; kidney-targeted delivery platforms are promising but remain preclinical [104].

### 4.3. Ferroptosis Inhibitors

**[Preclinical]** Ferroptosis—an iron-dependent, non-apoptotic cell death program—is posited to drive tubular injury in AKI. Canonical inhibitors ferrostatin-1 (Fer-1) and liproxstatin-1 (Lip-1) have been reported to block lipid peroxidation and preserve renal structure/function in multiple models [40]. Apolipoprotein-E appears to reduce ferroptosis by limiting ferritinophagy (the NCOA4-mediated degradation of ferritin) [153], while thiazolidinediones inhibit acyl-CoA synthetase long-chain family member 4 (ACSL4) and attenuate injury in glutathione peroxidase-4 (GPX4)-deficient settings [154]. More recent preclinical reports broaden the modulatory repertoire, including endocrine and nanomaterial-based interventions that dampen lipid-reactive oxygen species and restore nuclear factor erythroid 2–related factor 2 (Nrf2)/GPX4 tone; however, the evidence remains heterogeneous across cell-line systems and rodent ischemia–reperfusion or nephrotoxin models [155,156,157].

**Translation status:** No AKI-specific human interventional data yet; safety pharmacology (off-target antioxidant effects and chemotherapy interactions) and context selection (cisplatin, cardiopulmonary bypass/hemolysis-dominant states) will be critical for any proposed early-phase trials.

### 4.4. Hepcidin (Agonists/Mimetics)

Hepcidin limits iron egress by inhibiting ferroportin-mediated export; sequestered iron is incorporated into H-ferritin, theoretically reducing catalytic iron injury [113,114].

**[Preclinical]** Hepcidin or mimetics demonstrate protective potential in IRI and hemoglobin-mediated injury models [124,126]. **[Human]** Observational perioperative studies link higher urinary hepcidin to lower AKI risk [123,127]; associations with survival benefits have been reported in severe illness requiring dialysis [128]. **Translational status:** While hepcidin mimetics/agonists (e.g., minihepcidins and rusfertide) are clinically active in other iron-overload disorders [129,130,131], AKI-specific trials remain hypothesis-generating and should be biomarker-guided.

### 4.5. HO-1/Ferritin-H Induction

**[Preclinical]** Heme oxygenase-1 (HO-1) is characterized by antioxidant, anti-inflammatory, and anti-apoptotic effects [134] and degrades heme to biliverdin/bilirubin, CO, and Fe^2+^, which is then stored in ferritin—potentially limiting catalytic iron toxicity [158]. Induction of HO-1/FtH appears renoprotective across multiple injury models; however, **[Human]** interventional data in AKI are limited.

### 4.6. NGAL-Based Strategies (Engineered Variants)

Conventional iron chelators can sometimes disrupt tubular iron homeostasis—either over- or under-depleting iron—with potential functional consequences [159,160]. A conceptual alternative concept is to exploit endogenous, filterable iron-binding proteins to sequester and escort catalytic (labile) iron out of the nephron. Engineered NGAL (LCN2) variants have been reported in **preclinical** settings to potentially modulate siderophore/iron handling, but **no human interventional data** are available yet [148]; **Translational cautions:** any NGAL-based approach must account for NGAL’s context-dependent pro-inflammatory signaling, infection-related biology, and possible interference with clinical NGAL assays used for AKI diagnosis/prognosis.

**Translational synthesis and next steps:** The mechanisms outlined above converge on a practical question: where—and how—should iron-targeted approaches be tested in humans? Table 1 synthesizes the therapeutic classes (chelation, ferroptosis blockade, hepcidin–ferroportin modulation, HO-1/FtH induction, apo-transferrin, and NGAL-based concepts) against their putative mechanisms, model evidence, early human signals, and immediate translational steps. Table 2 then sketches conceptual, hypothesis-generating trial shells that align interventions to clinical contexts with the highest biologic plausibility (e.g., CPB/contrast exposure and cisplatin), pairing biomarker-guided enrichment (NGAL for injury detection; LPI/BDI, free hemoglobin, and TSAT for iron burden) with standardized endpoints (KDIGO AKI windows and MAKE30) to enable comparable readouts across studies. This layout is intended to move from pathway biology to testable designs without over-inferring efficacy from preclinical data.

## 5. Limitations

We acknowledge several limitations, including a dominance of preclinical evidence and a reliance on observational human studies. Furthermore, heterogeneous AKI definitions and clinical contexts, coupled with the limited standardization of catalytic iron (BDI/LPI) and hepcidin assays, complicate data synthesis and cross-study comparability. We also recognize methodological constraints—specifically single-reviewer screening and the absence of a formal risk-of-bias assessment—which further constrain causal inference and cross-study comparability; consequently, we emphasize that these findings are best interpreted as hypothesis-generating and confirmatory.

## 6. Conclusions

Iron is indispensable for renal metabolism, yet, when dysregulated, can drive oxidative and ER stress and ferroptotic injury. This narrative review integrates evidence across catalytic/labile iron, transferrin, ferritin/FtH–HO-1, the hepcidin–ferroportin axis, and NGAL in both preclinical and human AKI contexts.

Given the mechanistic heterogeneity of AKI, a single “silver bullet” is unlikely. Multimodal strategies that lower labile iron burden, modulate hepcidin–ferroportin signaling, augment ferritin/HO-1 defenses, inhibit ferroptosis, or leverage apo-transferrin/NGAL-based approaches may be required, tailored to clinical context. Iron-handling pathways are biologically plausible targets; however, current findings are hypothesis-generating. Near-term priorities include assay harmonization and feasibility studies before interventional testing.

Specifically, validation requires targeted, biomarker-guided trials that are needed in defined AKI settings (e.g., CPB, contrast exposure, cisplatin, rhabdomyolysis, and critical illness) using standardized assays (e.g., BDI/LPI for catalytic iron, hepcidin, and NGAL); clinically meaningful endpoints [64,65,66,67]; and explicit safety monitoring (anemia and infection) [103]. Until such evidence accrues, iron-targeted interventions should be considered investigational outside clinical trials; standard supportive care remains the foundation, with trial enrollment encouraged where available.

## Figures and Tables

**Table 1:** Therapeutic avenues targeting iron pathways in AKI.

Class	Candidate(s)	Mechanism of Action	Model/Phase	Human Data Status	Safety Notes	Translational Next Step
**Chelators**	DFO, deferiprone, EDTA	Bind catalytic iron; reduce ROS	Glycerol, cisplatin, IRI [20,22,79]	Small physiological studies [94]	Hypotension, infection risk, chelation toxicities	Pragmatic, biomarker-guided trials (CPB/contrast)
**Ferroptosis inhibitors**	Ferrostatin-1, Liproxstatin-1	Block lipid peroxidation (ferroptosis)	Multiple rodent AKI models [40,153,154,155,156,157]	No human data	Off-target/PK unknown in humans	Early-phase safety; cisplatin-AKI enrichment
**Hepcidin agonists**	Minihepcidins, rusfertide	↓ FPN export; sequester iron	IRI, hemoglobin-mediated models [124,126,131]	Observational only [123,127,128]	Hypoferremia, anemia	Pilot randomized controlled trials in hemolysis-dominant AKI [129,130]
**HO-1/FtH inducers**	NRF2/HO-1 inducers	↑ Heme degradation; ↑ ferritin buffering	Rodent AKI [134,158]	No human data	Off-target hemodynamics	Dose-finding; biomarkers (HO-1/FtH) [118]
**Transferrin strategies**	Apo-Tf	Limit iron delivery to viable cells	IRI-like models	No human data	Transfusion compatibility	Feasibility in CPB/contrast [103]
**NGAL-based**	Engineered NGAL variants	Siderophore/iron sequestration	Preclinical [148]	No human data	Interference with NGAL assays; inflammation	Mechanistic studies; patent-guided development

DFO: Deferoxamine; EDTA: Ethylenediaminetetraacetic acid; ROS: Reactive oxygen species; IRI: Ischemia–reperfusion injury; CPB: Cardiopulmonary bypass; AKI: Acute kidney injury; PK: Pharmacokinetics; FPN: Ferroportin; NRF2: Nuclear factor erythroid 2–related factor 2; HO-1: Heme oxygenase 1; FtH: Ferritin heavy chain; Apo Tf: Apotransferrin; NGAL: Neutrophil gelatinase-associated lipocalin. ↑ indicates increased/elevated levels. ↓ indicates decreased/reduced levels.

**Table 2:** Therapeutic avenues targeting iron pathways in AKI (conceptual, hypothesis-generating trial designs).

AKI Context	Intervention	Comparator	Enrichment Biomarkers	Primary Endpoint	Key Secondary	Sample-Size Signal *
**CPB-AKI prevention**	Apo-Tf peri-op **	Placebo	Catalytic iron (LPI/BDI) high, free Hb ↑, NGAL ↑	KDIGO AKI within 72 h	MAKE30; ICU length of stay	15–20% absolute risk reduction (ARR) *
**Contrast-associated AKI**	DFO short infusion	Placebo	Baseline LPI/BDI high	AKI within 48–72 h	Creatinine area under the curve; RRT	8–10% ARR *
**Cisplatin-AKI**	Ferrostatin-like agent **	Placebo	GPX4-low signature; NGAL ↑	AKI incidence over cycle 1	eGFR decline; chemo efficacy	10–15% ARR *
**Hemolysis-dominant ICU AKI**	Hepcidin agonist	Placebo	Free Hb ↑, TSAT > 60%, LPI/BDI ↑	RRT-free days	Mortality; infection	0.3–0.4 SD effect *

AKI: Acute kidney injury; CPB: Cardiopulmonary bypass; Apo Tf: Apotransferrin; peri-op: Perioperative; LPI: Labile plasma iron; BDI: Bleomycin-detectable iron; Hb: Hemoglobin; NGAL: Neutrophil gelatinase-associated lipocalin; KDIGO: Kidney Disease: Improving Global Outcomes; MAKE30: Major Adverse Kidney Events at 30 days; ARR: Absolute risk reduction; RRT: Renal replacement therapy; eGFR: Estimated glomerular filtration rate; GPX4: Glutathione peroxidase 4; TSAT: Transferrin saturation; SD effect: Standardized effect size (Cohen’s d). Note: * Proposed designs are conceptual; numerical figures are illustrating planning assumptions (not efficacy estimates). ** Human pharmacokinetic (PK)/dosing feasibility for Apo-Tf (apotransferrin) and ferroptosis inhibitors has not yet been established. ↑ indicates increased/elevated levels.

## Data Availability

No new data were created or analyzed in this study. Data sharing is not applicable to this article.

## Supplementary Materials

The following supporting information can be downloaded at: https://drive.google.com/drive/folders/14QpZ6fhmxyb6WJ4fiIAARtIQtOFvaQia?usp=sharing (accessed on 25 February 2026).

## Abbreviations

The following abbreviations are used in this manuscript:

ACSL4	Acyl-CoA synthetase long-chain family member 4
AKI	Acute kidney injury
Apo Tf	Apotransferrin
ARR	Absolute risk reduction
AUC	Area under the curve
BDI	Bleomycin-detectable iron
CPB	Cardiopulmonary bypass
DAMP	Damage-associated molecular pattern
DFO	Deferoxamine
EDTA	Ethylenediaminetetraacetic acid
eGFR	Estimated glomerular filtration rate
Fe^2+^	Ferrous iron
FPN	Ferroportin
FtH	Ferritin heavy chain
GPX4	Glutathione peroxidase 4
GR	Glutathione reductase
GSH	Reduced glutathione
GSSG	Oxidized glutathione (glutathione disulfide)
GSDMD/E	Gasdermin D/E
Hb	Hemoglobin
HO-1	Heme oxygenase 1
ICU	Intensive care unit
IRI	Ischemia–reperfusion injury
KDIGO	Kidney Disease: Improving Global Outcomes
LIP	Labile iron pool
Lipid-ROS	Lipid reactive oxygen species
LOS	Length of stay
LOXs	Lipoxygenases
LPI	Labile plasma iron
MAKE30	Major adverse kidney events at 30 days
NADP^+^	Oxidized nicotinamide adenine dinucleotide phosphate
NADPH	Reduced nicotinamide adenine dinucleotide phosphate
NCOA4	Nuclear receptor coactivator-4
NF-κB	Nuclear factor kappa-light-chain-enhancer of activated B cells
NGAL	Neutrophil gelatinase–associated lipocalin
NRF2	Nuclear factor erythroid 2–related factor 2
NTBI	Non-transferrin-bound iron
PK	Pharmacokinetics
pMLKL	Phosphorylated mixed-lineage kinase domain-like
PRR	Pattern-recognition receptor
RCT	Randomized controlled trial
RIPK	Receptor-interacting protein kinase
ROS	Reactive oxygen species
RRT	Renal replacement therapy
SD	Standard deviation
SF	Serum ferritin
SI	Serum iron
Tf	Transferrin
TfR	Transferrin receptor
TfR1	Transferrin receptor 1
TfR2	Transferrin receptor 2
TIBC	Total iron-binding capacity
TRF	Transferrin (concentration/index)
TSAT	Transferrin saturation
